# Video loss prediction model in wireless networks

**DOI:** 10.1371/journal.pone.0212407

**Published:** 2019-03-06

**Authors:** João Victor Costa Carmona, Edemir Marcus Carvalho de Matos, Bruno Souza Lyra Castro, Fabrício José Brito Barros, Miércio Cardoso de Alcântara Neto, Evaldo Gonçalves Pelaes

**Affiliations:** 1 Department of Computation, Federal University of South and Southeast of Pará, Marabá, Pará, Brazil; 2 Department of Computation, Federal University of Pará, Belém, Pará, Brazil; Nanjing University of Information Science and Technology, CHINA

## Abstract

This work discusses video communications over wireless networks (IEEE 802.11ac standard). The videos are in three different resolutions: 720p, 1080p, and 2160p. It is essential to study the performance of these media in access technologies to enhance the current coding and communications techniques. This study sets out a video quality prediction model that includes the different resolutions that are based on wireless network terms and conditions, an approach that has not previously been adopted in the literature. The model involves obtaining Service and Experience Quality Metrics, such as PSNR (Peak Signal-to-Noise Ratio) and packet loss. This article outlines a methodology and mathematical model for video quality loss in the wireless network from simulated data and its accuracy is ensured through the use of performance metrics (RMSE and Standard Deviation). The methodology is based on two mathematical functions, (logarithmic and exponential), and their parameters are defined by linear regression. The model obtained RMSE values and standard deviation of 2.32 dB and 2.2 dB for the predicted values, respectively. The results should lead to a CODEC (Coder-Decoder) improvement and contribute to a better wireless networks design.

## 1 Introduction

According to CISCO reports [1, 2], a significant increase in video traffic has been observed on the internet. Developers and researchers will have to address this demand in the years ahead. There has been an increase in the flow of multimedia information resulting from the popularization of wireless networks and the rise in the use of mobile devices with connections that also follow this network pattern [3]. Moreover, the limitations of the wired medium, such as user mobility and its physical structure [4] for deployment, has led to an extension of the standards for the wireless network. In addition, the growing number of users accessing the internet, has resulted in alternative systems for the distribution of data, audio, and video services between the subscribers.

In wireless networks, the most widely used technology is IEEE 802.11 [5], which was developed by the Institute of Electrical and Electronic Engineers—IEEE. In this “new” era of technology, applications provide special benefits, and make improvements that enhance service quality and end-user experience.

The standard 802.11ac was first applied in 2008 and operates in the 5 GHz band; it relies on two basic concepts/assumptions unlike other versions (such as a, b, g, n). This standard employs the Multiple Input and Multiple Output (MIMO) technique, and higher channel bandwidth. There has been a rapid growth in the use of video streaming because of the improved Internet connection speed [6]. Thus it is essential to, study the performance of the 802.11ac standard for a video streaming service so that the video quality loss can be estimated.

The increased video streaming in last-mile technologies, especially WLANs, has required high transfer rates resulting from alterations in end-user profiles, which have led to the consumption of high and ultra-high-resolution video. One type of access technology employed to satisfy this demand, is IEEE 802.11ac, which is regarded as the “last mile” access technology candidate that can support the video streaming service at high and ultra-high resolutions to provide a better quality of service and experience [7]. Hence, modeling the video quality loss in this technology is critical from the standpoint of both service and the end-user experience [8].

The ability of high-resolution videos to provide people with an improved quality of experience is beginning to attract a good deal of attention because this type of service is used by a large number of users. Thus, coding is in progress as well as the optimization of network planning. In the case of coding, the objective is to provide acceptable video quality, and hence reduce the storage space for information [9]. Both quality of experience and quality of service are included in the network planning [10].

This work discusses the video transmitted by the standard IEEE 802.11ac wireless communications network, and designs a model to predict the video quality loss caused by packet loss. This model is based on simulated and subsequently validated data based on video measurements in a real transmission environment. In the literature, mathematical modeling is not usually employed to predict the loss of quality of videos, since solutions can usually be found in computational intelligence. Moreover, in [11–13], methodologies were devised to predict the video quality using computational intelligence techniques. In this paper, a mathematical model is put forward for the prediction of video quality loss in a wireless network. Four videos are included in the modeling in three resolutions and encoder H.264/AVC with a constant bit rate. The data for model development are obtained from extensive simulations network packet loss. The authors validated the scheme by making measurements in the real environment and obtaining performance values for the model, such as RMSE and Standard Deviation. This demonstrated that the predictive model for video quality loss is necessary.

The main contribution made by this article is to design a methodology and mathematical model to predict the video quality loss in a wireless network. As far as was verified by the research of the authors, no studies were found that recommend the use of mathematical models to predict video quality loss in wireless networks.

This work consists of an introduction and five further sections. Some related work is examined in section 2. section 3 outlines the problem and the methodology adopted to employ the model for predicting the video quality loss. section 4 discusses how real data is obtained and how they are handled statistically for the validation of the model. section 5 discusses the performance of the model and its restricted use. Finally, the conclusions are summarized in section 6.

## 2 Related work

There is a real need to adopt new approaches to Quality of Experience based on Quality of Service. In [14], two Quality of Experience metrics are correlated on the basis of Quality of Service. In this study, an estimate is made of the loss of packets for twelve different videos in CIF and 4CIF formats, by means of the following metrics: Peak Signal to Noise Ratio (PSNR) and Mean Opinion Score (MOS). Correlation values are obtained that are higher than 0.95 and between PSNR and MOS to prove that the PSNR metric is sufficiently reliable to represent the degree of degradation in video quality. However, the experimental results cannot be validated with measures in a real environmental transmission, in addition to failing to take account of videos in high and ultra resolution.

In [15], the strategy is focused on exploring the source encoder. The authors employ a model to estimate video quality without using the original video as a reference-point, and the solution is added as a part of CODEC H.264 that includes the metrics of the source encoder. The values obtained, that are based on the developed model, are compared with the SSIM objective metric. Two performance metrics can be employed to assess the results: the Pearson Correlation Coefficient (PCC) and Root Mean Square Error (RMSE). This study concludes that the model to assess video quality without a reference-point, is as reliable as SSIM. This system is of value for video streaming because the Quality of Service of the communication network directly influences the Quality of Experience. However, the authors only explore one of the factors that cause video degradation and disregard the terms and conditions of the communication network and resolution.

In [16, 17], the authors recommend a more efficient coding system, by assessing the performance of video processing in several resolutions based on simulations. They used metrics such as Speedup and PSNR in environments simulated with several coreprocessors. As well as this, in [17], the Motion Estimation (ME) is carried out by means of execution partitioning for specific tasks like video coding. Thus, it was possible to accelerate the video encoding process without causing damage, and as a result, the procedure was approximately ten times faster than any of the other methods. This underlines that there is a lack of studies using real environments.

Another quality analysis involves the use of video streaming to make adjustments to the unfavorable conditions for transmission (in the communication channel). The authors in [18] studied a client-server video streaming environment, with the aim of monitoring the network conditions, as well as the buffer state in the communications system of 4G LTE mobile networks. On the basis of the buffer analysis, the authors devised a methodology to estimate the video quality received by the client by following some Quality of Experience metrics: PSNR, SSIM, and Video Quality Measurement (VQM). The client can draw on the estimates to inform the server if there is any degradation in the video transmitted and allow adjustments to be made by the server. The changes in the video entail modifying the resolutions in time. The algorithm that was created achieved good results when estimating the degree of video degradation and changing the video resolution to improve its quality. Although the study includes Robust Communications Software (4G LTE), the video resolutions do not reflect the users’ requirements for video streaming, since the study resolutions are not between high and ultra resolution.

On the basis of this study and other works like [12, 19], in which techniques are devised to predict image patterns in intelligent vehicles, as well as in [16, 17, 20–22], the authors are able to suggest improvements in source coding to optimize the performance of these systems. In view of this, it is necessary to optimally map the packet loss, with application layer features (PSNR and MOS), which some authors regard as a Cross-Layer approach [23, 24].

From the above in the preceding paragraphs, there is a deficiency in research based on mathematical formalism to predict aspects of QoE from the QoS monitoring. The authors of [25] present a generic mathematical expression that relates QoE with QoS for Voice over IP (VoIP). However, the user profile multimedia content has changed, which makes use of videos in high resolution [7]. Therefore, studying the adoption of mathematical expression to predict the video quality loss is fundamental to improve the video streaming service in wireless networks.

## 3 The proposed model

It is necessary to check the performance of the videos transmitted when designing a video loss prediction model, based on the communication network standards (IEEE 802.11ac). A set of techniques is required such as simulation, modeling, and measurement. The modeling process uses computational tools such as the EVALVID framework [26] to transmit and reconstruct videos and the MATLAB software to simulate and handle information.

The next subsections will describe in detail the methodology employed in this work to carry out the task of designing a video loss prediction model.

### 3.1 A problematic area

Wireless networks are prone to error owing to some of the features of the communication channel, such as trajectory loss, diffraction, and refraction. These phenomena directly affect the quality of service when the video streaming over the wireless network is analyzed. It is necessary to evaluate user satisfaction by means of quality of experience metrics. For this reason, this work evaluates the transmission of videos in high and ultra-resolution in the wireless network. This study also provides a model that is capable of predicting the video quality loss.

It is necessary to design predictive models that relate QoS and QoE metrics to specific video streaming applications in wireless networks, as discussed in the introduction and related work sections. These models can complement the development of QoE-driven wireless access technologies, as well as making improvements to the source encoders.

### 3.2 Generation of reference data

The process of data collection constitutes the first stage in carrying out the study and modeling. An “ideal transmission” was conducted in a controlled environment, i.e., a lossless, simultaneous transmission between just two dedicated computers. Alterations were made to the original file with the aim of obtaining an encoded video file without losses, in which the packets transmitted would be equal to the amount received, i.e., some lines forming this file were removed, thus reducing its size and, hence, the number of packets.

Thus, in this controlled environment (i.e. the simulation), a total of twelve transmissions were transmitted to four types of videos, in which checks were made of the packages sent by the server´s computer and the packages received by the client’s computer. The result was that the files which included the number of sent packets, was the same as the number received, and this faithfully represented each video.

The number of packages sent and received during the stage of video transmission was determined by using the TCPDUMP tool, which, after selecting the send and receive port, captures the packages that have to be analyzed. In this way, the number of packages that are sent and received in each transmission, is obtained and saved in the file [27].

**Fig 1:**
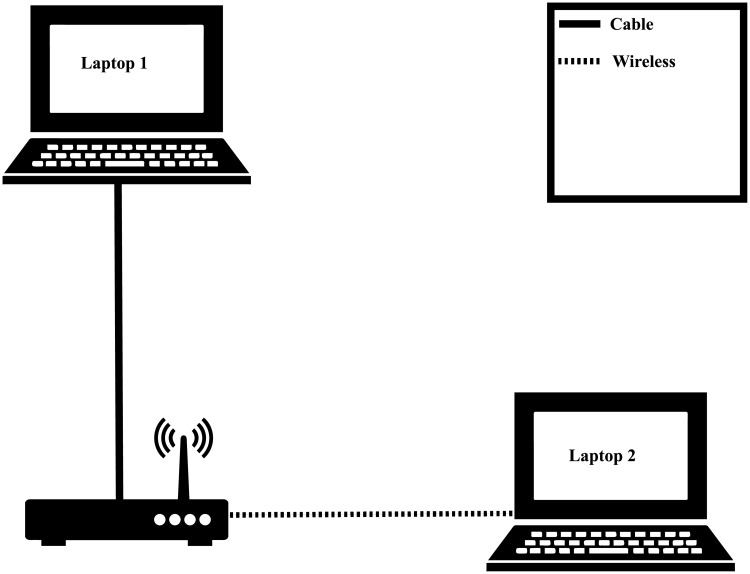
Illustration of video transmission and reception.

Thus, a scenario was set up: this involved two notebooks (client and video server) connected to an access point (see Fig 1 below). The communication interface between Notebook 1 and the Access Point is wired (to reduce the chances of packet loss while the video is being sent), and the communication interface between the Access Point and Notebook 2 is wireless (IEEE 802.11ac). Note that the scenario is used to obtain the original data (without packet loss) so that it can be used both to simulate and obtain data in the real environment.

Fig 1 shows a typical streaming environment, where Notebook 1 streams video to Notebook 2. The access point represents the equipment using the wireless standard 802.11ac, that provides the communication interface between both terminals.

The videos were obtained from a multimedia base called “Xiph.Org Foundation” (The videos are available on https://media.xiph.org/video/derf/) in three different resolutions: 720p, 1080p, 2160p (4k). The names of the videos used are: (a) Crowd Run, (b) Ducks Take Off, (c) Park Joy, and (d) IntoTree. A significant factor that differentiates the videos is the number of motions throughout their reproduction, where videos with small, average, and intense variations in motion are used.

Table 1 shows the setting information of the CODEC parameters. Each video consists of 500 frames. Thus, in this work, each video will be reproduced for 10 seconds. Any adjustment in the coding parameters will directly influence the level of video quality.

**Table 1:** Source encoder setting.

Encoder	H.264/AVC
**Bit rate**	32 Mbps
**GOP**	25
**FPS**	50

In this scenario, there were twelve transmissions to four videos (for three resolutions) and the packages sent by the server with the packages received by the client were analyzed. If the number of packages sent and received was equal (see Table 2), it was assumed that there was no loss. The file manipulation process is described below.

**Table 2:** Number of packages per transmission.

		Number of packages	
Vídeo	Resolution	Sent	Received
**Crowd Run**	**720p**	40361	40361
	**1080p**	40562	40562
	**2160p**	40875	40875
**DucksTake Off**	**720p**	40488	40488
	**1080p**	40674	40674
	**2160p**	43199	43199
**Park Joy**	**720p**	39488	39488
	**1080p**	40003	40003
	**2160p**	40068	40068
**In to Tree**	**720p**	41544	41544
	**1080p**	42424	42424
	**2160p**	44754	44754

### 3.3 Simulation

The wireless communication channel is essentially random, so in this work, it was assumed that the relationship with packet loss can be described by a uniform and have a discrete distribution. On the basis of the coding files with no video loss, that were obtained in the preceding stage, the probability mass function was applied to define the lines that had to be removed from the received file, as in Eq 1 [28]:

$$\begin{array}{c} f\left(x\right)=\{\begin{array}{c} \frac{1}{n},\;if\;1\leq x\leq n;\\ 0,\;otherwise \end{array} \end{array}$$ (1)

where “n” represents the total number of packages of a certain video (the maximum values can be seen in the column received from Table 2). Thus, there is the same probability for all the packages comprising the video that they will be removed from the encoded file, which is obtained through TCPDUMP.

**Fig 2:**
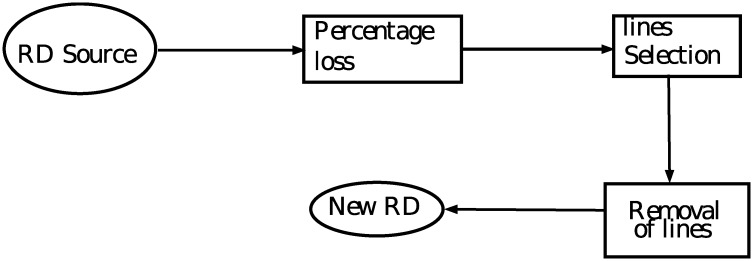
Dragging mechanism for the lines removed from the encoded video file.

Thus, the routine to simulate packet loss was established where certain lines of the source file could be basically removed (“RD Source”). The stages followed by the algorithm are: a) setting the initial loss at 2%, which represents the total number of lines to be removed, and b) adding 2% to that value up to 30%. This routine applies to each set of video in the files that correspond to the receipt of the file. Thus, there will be 15 new encoded files for each video, with losses, which now have different percentages. The procedure to remove the line is outlined in Fig 2, where: “RD Source” is the original file containing the transmitted video traces, “Percentage loss” is the loss variation (of 2% to 30%), “lines Section” are the lines to be removed, “Removal of lines” is the function for removing the lines of the original file, “NewRD” is the new file (changed).

It is worth emphasizing the importance of this removal routine. Several files are created that correspond to the degraded videos are, after their application, and have a packet loss rate. These files are of crucial importance to extract the PSNR metric (in dB) of the simulated transmissions based on the percentage of packet losses. These values are obtained by reconstructing the video received through the EVALVID software [29]. In total, there were 50 simulations for each type of video, resulting in 9,000 samples with losses, 750 of which are for each video that is definitely linked to a certain resolution (12 types of video).

### 3.4 Modeling methodology

#### 3.4.1 Simulation: PSNR loss vs. packet loss

One of the most mathematical methods used to measure video quality is PSNR, which is based on statistical parameters for images. It defines the relationship between the potential maximum power of a signal and the noise affecting the representation of this signal between the original and degraded video frames.

PSNR is the objective metric used to evaluate the video quality received by the user compared with that of the original video. It is measured in a logarithmic scale and depends on another metric called Mean Squared Error (MSE) to estimate the difference between the original video and the video received [30]. The MSE and the PSNR are defined by Eqs 2 and 3, respectively:

$$\begin{array}{c} MSE=\frac{1}{MN}\sum_{i=0}^{M-1}\sum_{j=0}^{N-1}{\left[I_{\left(i,j\right)}-K_{\left(i,j\right)}\right]}^{2} \end{array}$$ (2)

where:

MSE—*Mean Squared Error*.

*I*_(*i*,*j*)_—matrix that forms a transmitted frame.

*K*_(*i*,*j*)_—matrix that forms a received frame.

*M* and *N*—represent the dimension of the frame.

$$\begin{array}{c} PSNR=10{\text{log}}_{10}(\frac{{\left(2^{m}-1\right)}^{2}}{MSE}) \end{array}$$ (3)

where (2^*m*^ − 1)^2^ represents the frame of the highest possible value of a pixel in the image, where n is the number of bits required to represent a pixel of the image.

Table 3 referencia shows the maximum PSNR values of the original videos (degraded). These values are useful to determine the variations in the quality loss of the videos used in this study. The PSNR loss was analyzed to assess the impact of the packet loss on the videos, first in a simulated environment and later in a comparison with the actual losses in the second scenario (validation). Thus, the values in Eq 4 are used to determine the PSNR loss, and the values in Table 3 are used for the *L*_*ref*_ variable Note that, as the video consists of a set of frames and the EVALVID provides us with the PSNR value per frame, the average mean PSNR value of the video was taken to represent the video quality.

$$\begin{array}{c} L=L_{ref}-L_{sim} \end{array}$$ (4)

Where:

*L*—mean PSNR loss in *dB*;

*L*_*ref*_—mean PSNR values of the video transmitted in *dB*, without losses;

*L*_*sim*_—mean PSNR value of the video received in *dB*.

**Table 3:** Average mean PSNR per video.

	PSNR (dB)		
Name of Video	720p	1080p	2160p
Crowd Run	38.45	35.82	31.66
Ducks Take Off	36.22	30.69	27.4
Park Joy	37.7	31.58	30.13
In to Tree	40.97	36.93	34.3

**Fig 3:**
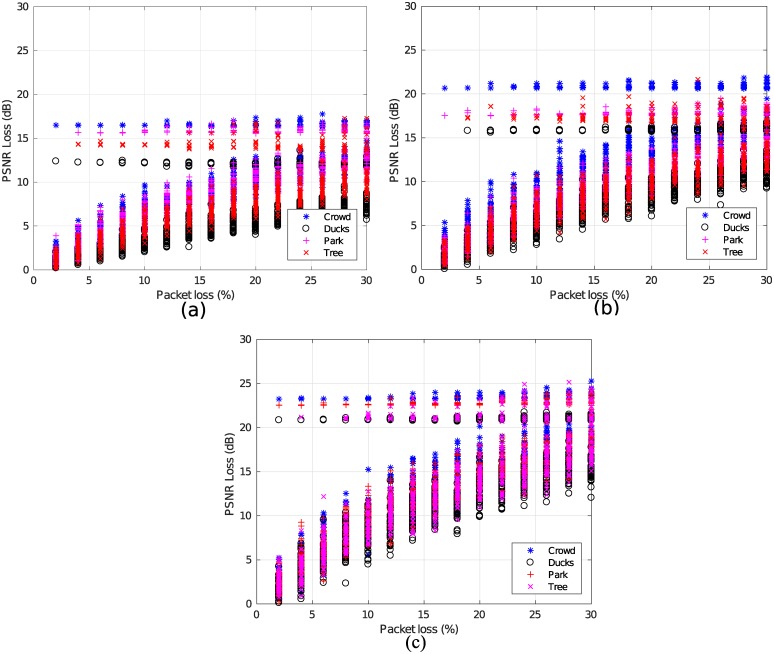
Simulated data. (a) For 2160p, (b) For 1080p and (c) For 720p.

In Fig 3, the results of the PSNR loss values (obtained after the application of Eq 4) are determined as a function of the packet loss in the simulations for each video resolution. Fig 3(a) shows the PSNR loss values of four types of videos based on the percentage of packet loss, where the packet loss increases the PSNR loss. The same response can be observed in Fig 3(b) and 3(c). It should however be noted that in the results of the simulations, the existence of PSNR loss values do not describe the degree of quality degradation. These points are analyzed in subsubsection 3.4.2.

In the graphics, it can be seen that there are videos that are more susceptible to the loss of packages when the loss of PSNR is analyzed. This feature was determined by disregarding the resolution, and concluding that there are features of the type of video (mainly movement) that can influence the performance of the encoder during the transmission. This is probably due to the loss of P frames, which includes information on motion compensation [31–33].

#### 3.4.2 Modeling: PSNR loss vs. packet loss

In simulated data, there are points that do not describe the loss of PSNR (Outliers), which means that they can be identified and removed. The standard deviation was used, while adding the upper cutting limit and subtracting the lower cut-off limit of the standard deviation of the samples, as expressed in Eq 5 [28].

In simulated data, there are points that do not describe the loss of PSNR (Outliers), so they are identified and removed. The standard deviation was used, adding the upper cutting limit and subtracting the lower cut-off limit of the standard deviation of the samples, according to Eq 5 [28].

$$\begin{array}{c} L_{i}=\{\begin{array}{c} outlier,\;L_{fit}-D_{p}>Li>L_{fit}+D_{p}\\ no,\;otherwise \end{array}\} \end{array}$$ (5)

where:

*L*_*fit*_—represents the mathematical function of the data;

*D*_*p*_—is the standard deviation of data.

Thus, all the data for each resolution will be grouped and combined with a certain percentage of packet losses, with the aim of identifying the outliers. For example, the results (irrespective of the video) of the 720 p simulations provide all the values corresponding to 2% of the packet loss. Later, the outliers will be identified and removed through analysis and the other resolutions will follow this same procedure.

**Fig 4:**
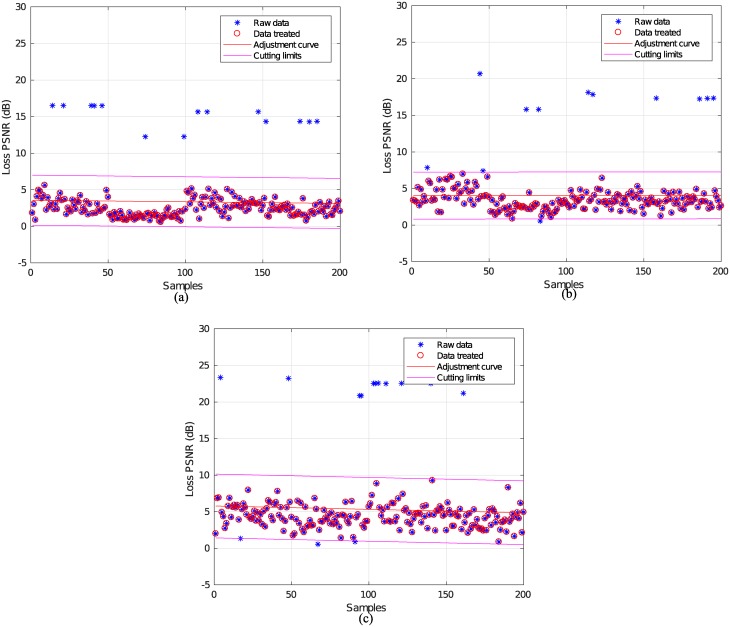
Data handling for packet losses of 4%. (a) 2160p, (b) 1080p e (c) 720p.

The best estimate for describing the simulated data is the first degree polynomial function. After obtaining the mathematical representation, the standard deviation is applied to these samples and the upper and lower limits can be defined for data filtering, as displayed in Fig 4:

After this filtering, a total of 37 samples of the three resolutions were rejected (see Fig 4). On average, in this study, 76% of the simulated data were used to estimate the PSNR loss based on the packet loss percentage.

The PSNR loss values for the four videos are known for each resolution, and the strategy was employed of grouping their values by resolution. Therefore, for each study resolution, the average loss of PSNR of the four videos is obtained as a function of the values of the packet losses, using Eq 6. The analysis of the proposed model will be based on these loss values.

$$\begin{array}{c} L_{simu}\left(PL\right)=\frac{\sum_{k=1}^{F}L_{r}\left(k\right)}{F} \end{array}$$ (6)

where:

*PL*—is the packet loss percentage;

*L*_*r*_—is the PSNR loss;

*F*—is the value of the number of samples.

After filtering the data, the best expression to represent the PSNR loss was obtained. As a result, a logarithmic function was defined by (Eq 7).

$$\begin{array}{c} L_{PSNR_{fit}}=a+blog_{10}\left(PL\right) \end{array}$$ (7)

where:

*PL*—is the packet loss percentage;

*a* and *b*—are coefficients.

To obtain the expression above, the mathematical formula of the Minimum Squares Method [34] was used, which is expressed as:

$$\begin{array}{c} A=[\begin{array}{cc} 1 & log_{10}\left(PL_{i}\right)\\ 1 & log_{10}\left(PL_{i}\right) \end{array}];\;coef=[\begin{array}{c} a\\ b \end{array}];\;B=[\begin{array}{c} L_{1}\\ L_{2} \end{array}];\;coef={\left(A^{\prime }A\right)}^{-1}A^{\prime }B \end{array}$$ (8)

Thus, a and b coefficients were obtained for each type of resolution (720p, 1080p e 2160p). An identical method was used to determine the general expression for estimating the PSNR loss as a resolution function and related packet loss. However, the resolution-related objective function depends on a decreasing exponential function.

**Fig 5:**
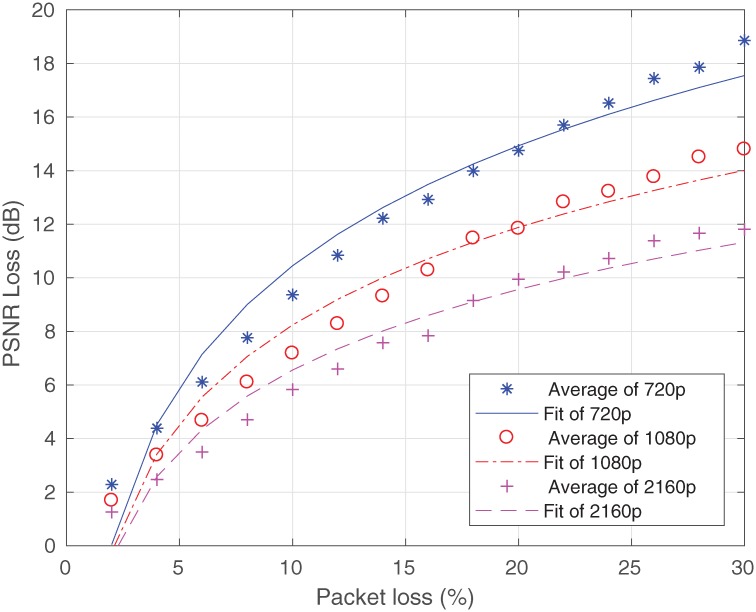
Mean average in data handling vs estimates.

In Fig 5, the graphs show the behavior of the three video resolutions. These curves were used to find the values of the expression coefficients of each video resolution in accordance with Eq 7.

As a result of the application of the general equation, the following expressions were found with their appropriate coefficients:

$$\begin{array}{c} L_{720p}=25.32+14.88log_{10}\left(PL\right) \end{array}$$ (9)

$$\begin{array}{c} L_{1080p}=20.34+12.1log_{10}\left(PL\right) \end{array}$$ (10)

$$\begin{array}{c} L_{2160p}=16.55+10log_{10}\left(PL\right) \end{array}$$ (11)

where:

*PL*—is the packet loss percentage.

*L*_720*p*_—is the approximation for the loss values of PSNR for the resolution of 720p;

*L*_1080*p*_—is the approximation for the loss values of PSNR for the resolution of 1080p;

*L*_2160*p*_—is the approximation for the loss values of PSNR for the resolution of 2160p.

Thus, it should be noted that at high resolutions (2160p and 1080p, with packet losses of up to 2.25%), there was no significant loss of PSNR for the end user, i.e. the impact was very small. This means that the high and ultra-resolution are more robust than the loss of packets to the constant bit rate value of 32 Mbps.

As the equations are based on the logarithmic function, when differentiated by the coefficients (a and b), and since each resolution has an equation (Eqs 9, 10 and 11), it can be concluded that its coefficients are related to the resolution.

At this stage, an analysis must be made to determine the coefficients a and b. Initially, the values are determined in terms of the variable related to the resolution. In this study, the resolution will be represented by Rand and can assume the following values: one (1), one and a half (1.5) and three (3) for 720p, 1080p and 2160p, respectively. In this case, the expression that best describes the coefficient is the exponential (Eq 12), as both decline when there is an increased resolution (see the coefficients in Eqs 9, 10 and 11 in Table 4).

$$\begin{array}{c} C=D+Ge^{\left(-R\right)} \end{array}$$ (12)

where:

*C*—is the function to analyze the data;

*D* and *G*—are constant;

*R*—is the type of resolution.

**Table 4:** Variable a and b.

	Resolution		
Variables	720p	1080p	2160p
**Value of a**	25.32	20.34	16.55
**Value of b**	14.88	12.1	10

The expressions defining a and b are set out in Eqs 13 and 14 to estimate the variables a (*C*_*a*_) and b (*C*_*b*_), respectively.

$$\begin{array}{c} C_{a}=14.89+27.4e^{\left(-R\right)} \end{array}$$ (13)

$$\begin{array}{c} C_{b}=9.07+15.24e^{\left(-R\right)} \end{array}$$ (14)

where:

R—is related to the resolution (values 1, 1.5, and 3).

With the aid of the simulated data and data handling, it is possible to describe the loss of PSNR, as there are packet losses for each resolution. This expression is:

$$\begin{array}{c} L_{PSNR}=C_{a}+C_{b}{\text{log}}_{10}\left(PL\right) \end{array}$$ (15)

where:

*PL*—is the packet loss percentage;

*C*_*a*_ and *C*_*b*_—are given by Eqs 13 and 14, respectively.

Expanding the equation above to cover the three video resolutions under examination:

$$\begin{array}{c} L_{PSNR}\left(R,PL\right)=\left(14.89+27.4e^{\left(-R\right)}\right)+\left(9.07+15.24e^{\left(-R\right)}\right){\text{log}}_{10}\left(PL\right) \end{array}$$ (16)

where:

*L*_*PSNR*_(*R*, *PL*)—is the PSNR loss estimate, in *dB*;

R—is the type of resolution (values 1, 1, 5, and 3)

*PL*—is the packet loss percentage (%).

The equation above estimates the PSNR loss caused by the packet loss and video resolution. The results of the equation when applied to the simulated data are shown in Table 5, in Standard Deviation and Root Mean Square Error (RMSE):

**Table 5:** RMSE and standard deviation of the model for the simulated data.

	Resolution		
Metric	720p	1080p	2160p
**RMSE in *dB***	1.3	0.89	0.73
**Standard Deviation (in dB)**	0.97	0.85	0.74

Note that, (for the three resolutions), the approximate RMSE value is, on average, 0.97 dB and 0.29 dB of standard deviation. These are satisfactory results for the model obtained when based on the linear regression. The validation of the model will be compared with the real environment measurements and shown below.

## 4 Measurements

Realistic video data were obtained by conducting measurement campaigns, in which the video was transmitted over the WLAN standard. These data will be used to validate the methodology employed for modeling the video quality loss in the network.

**Fig 6:**
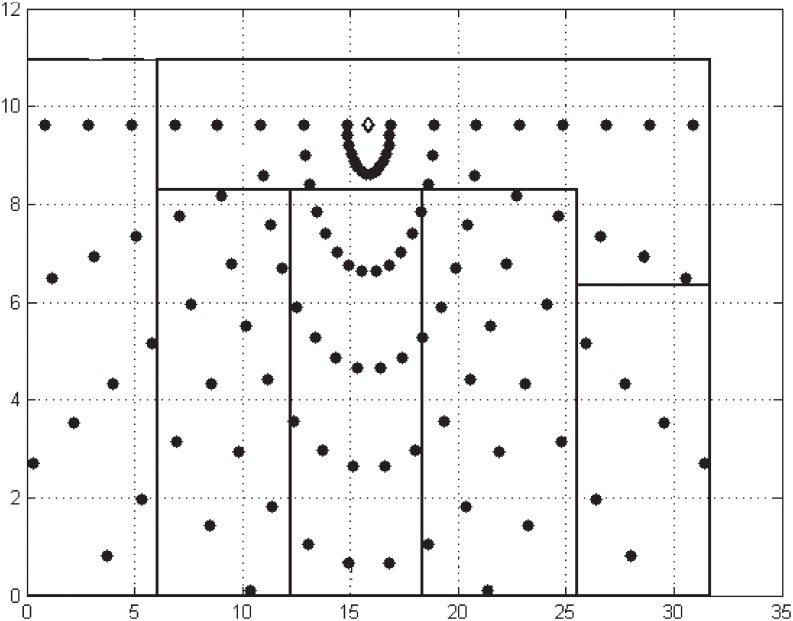
The measurement scenario.

In this case study scenario, the measurement points were arranged in 16 radials around the focal point (access point). The points for each radial, were spaced about 2 meters apart and, at every point, three video transmissions were carried out of Crowd Run, and resolution 2160p in wireless IEEE 802.11ac standard. The measurement system was standardized to ensure there were uniform measured points and to enable the use of a greater area of the scenario [35]. Fig 6 shows the plant of the room in Building B of the Institute of Technology at the Federal University of Pará, where the measurements were carried out, with the aim of validating the model.

It was noted that, as the access point operates in the 5 GHz frequency band, some points that were far from the access point were disconnected which prevented data from being collected. This was due to the signal fading caused by the number of masonry walls. Owing to the fact that the wavelength is shorter than the standard working frequency (5 GHz), the distortions along the course tend to be higher, and so the 802.11ac network lacks a suitable coverage for the case study scenario. As a result, the effects on the videos transmitted at these points were so harmful that they prevented their reconstruction and, hence, no metrics were obtained to validate the video quality.

**Fig 7:**
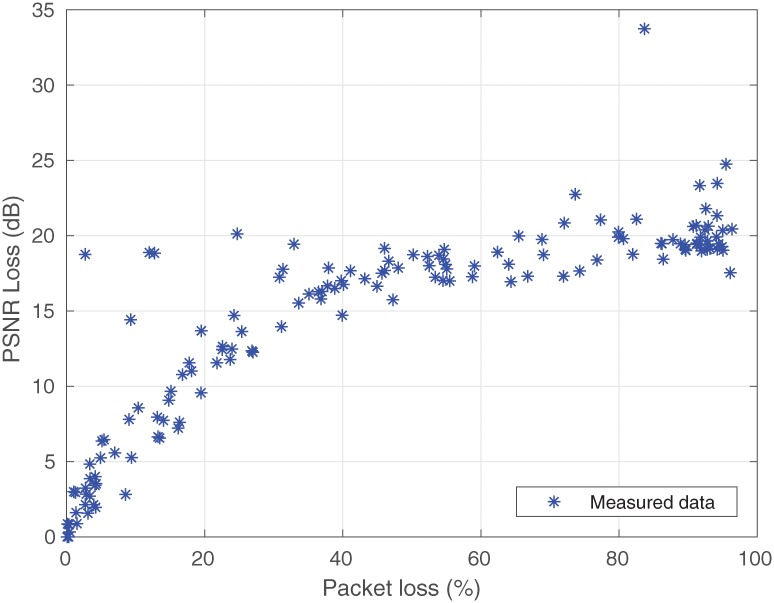
Data measured.

The transmissions in real time were made with the “CrowdRun” video in resolution 2160p, encoded with H.264/AVC, in standard 802.11ac (Fig 1), which follows the same stages as the transmissions for this work, except for a change in the bit rate used to encode the video, which is 82 Mbps. After the measurement campaign in the scenario (Fig 6), the PSNR loss values were obtained for this video, which are shown in Fig 7.

**Fig 8:**
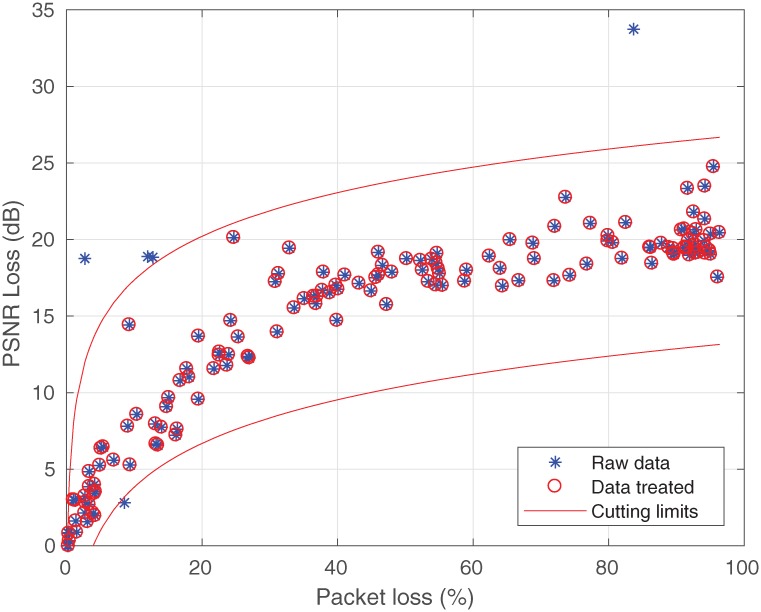
Removal of the measured data.

The outliers were also identified in the measurement, which was similar to that carried out in the simulation process. These samples do not represent the PSNR loss that is based on information about the standard deviation. Samples (after the measurement processing) are shown in Fig 8 below.

The values obtained after the elimination of the outliers will be used to make a comparison with the values predicted by the model recommended for this work.

## 5 Results and discussion

The analysis of the PSNR loss of the transmitted videos (only for resolution 2160p) suggests that there is a connection between the video quality loss and the packet loss (see Fig 8). In addition, it was possible to corroborate the report in the study [36] which confirmed that there really is an influence on the variation of the bit rate. Thus, a gain of 2.87 dB was added to the loss prediction model, resulting in Eq 17, where R is replaced by the value of 3.

$$\begin{array}{c} L_{PSNR}\left(3,PL\right)=\left[14.89+27.4e^{\left(-3\right)}\right]+\left[\left(9.07+15.24e^{\left(-3\right)}\right)\text{log}10\left(PL\right)\right]+2.87 \end{array}$$ (17)

**Fig 9:**
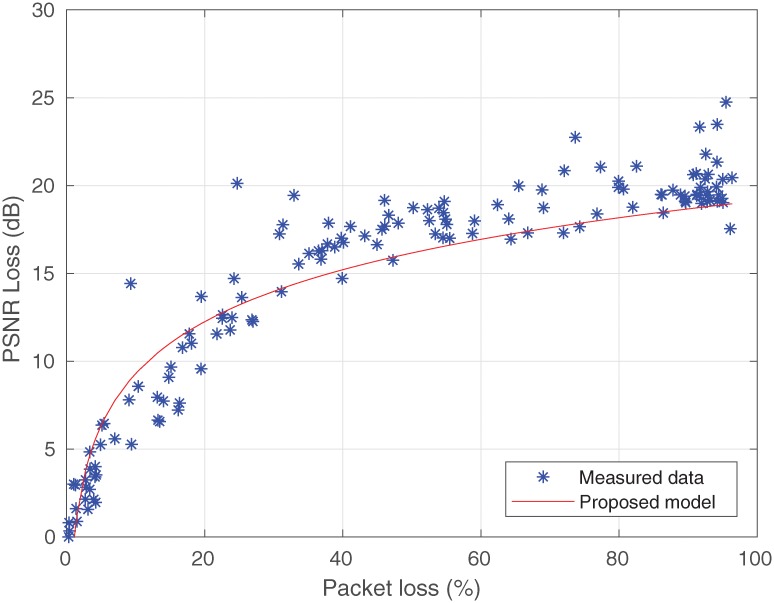
Recommended model for the measured data.

The values estimated by Eq 17 are shown in Fig 9 and compared with their measured values. It should be noted that the choice of the logarithmic function to model the video quality loss was well represented. From this result, it can be concluded that a bit variation rate can influence the pattern of PSNR loss, and confirms the decision to add the value of 2.87 to Eq 17. Thus, there is an opportunity for studies to add different bit rate values to this model.

It could be noted in Fig 9 that the model obtained satisfactory values, which are close to the measured data when based on a graphic analysis. The results of this analysis were validated by the RMSE model and by calculating the standard deviation, which were about 2.32dB and 2.2 dB, respectively.

There are no studies in the literature to assess the strategy of predicting the video quality loss on the basis of the number of packets or by using simple mathematical mechanisms. In light of this, techniques to reduce packet loss were studied in [13, 37]. However, these approaches failed to take account of the real packet loss during the video transmission in communications network 802.11ac, which makes this work unique in this respect. The results shown in this study, provide evidence that this line of research is a promising way of improving the coding techniques, as well as wireless planning. However, the model is restricted to the sourcecoding parameters defined in this work (Table 1) and thus cannot be tested for different Quantization Parameters (QP). The methodology employed in this study can be used for other types of video and sourcecoding; moreover, it might be possible to improve the model, since it is based on mathematical tools.

## 6 Conclusion

This study has set out a model to predict the video quality loss in a wireless communications network. This led to a simulated packet loss, but subsequently were obtained in a real environment during the video transmission. The same source coding was applied to standard H.264/AVC, although they were set with different resolutions for later transmission in an IEEE 802.11ac network. An ideal flow (without packet loss) was obtained for the files that are required to reconstruct videos. The mathematical model is used for the following: a) Simulating the packet loss on reception, b) reconstructing and obtaining video quality metrics, and (c) handling the simulated data. Additionally, the model links the quality loss and the packet loss in the three study resolutions.

From the results obtained from the simulations, it can be concluded that in video transmissions, the video quality loss may be subject to variations while there is a loss of packets, depending on the characteristics of their movement (low, medium or high). This observation, together with the CODEC parameters, such as bit rate and GOP size, will be investigated in future work.

The model recommended here is based on the average patterns of PSNR losses in the studied videos, according to the quality loss of four videos in three resolutions. The leasts mean squares method applies to three means of obtaining relevant estimates through resolution. The mathematical expression that depends on video resolution, is obtained through the estimates, which describes the prediction of video quality loss based on the packet loss. In general, the logarithmic function describes the average mean of the PSNR loss. The coefficients of the logarithmic function are described through an exponential function based on the type of resolution. The methodology used in this work for the modeling strategy takes the form of a simple implementation at low computational cost, which can be used in both wireless network planning and improving source encoders (CODEC).

The model recommended here is robust and achieved good results when compared with the mean simulated data, since it obtained a mean RMSE value of 0.97dB and standard deviation of 0.85dB. The equations derived were applied to real data to check the effectiveness of the model, i.e. a mean average RMSE of 2.32 dB and standard deviation of 2.2 dB were obtained for the data collected during an indoor measurement campaign. Thus, the results are satisfactory.

As far as the authors searched it is not usual to apply mathematical formalism to develop the predictive model. As a result appears a mathematical expression can predict video quality loss values in wireless networks. Measurements are compared to evaluate the model’s performance, which proved to have good results. It is believed that the results of this study may help in the improvement of source encoders and streaming video services.
